# VEGF Polymorphism rs3025039 and Human T-Cell Leukemia Virus 1 (HTLV-1) Infection among Older Japanese Individuals: A Cross-Sectional Study

**DOI:** 10.3390/bioengineering9100527

**Published:** 2022-10-06

**Authors:** Yuji Shimizu, Hirotomo Yamanashi, Jun Miyata, Midori Takada, Yuko Noguchi, Yukiko Honda, Fumiaki Nonaka, Seiko Nakamichi, Yasuhiro Nagata, Takahiro Maeda

**Affiliations:** 1 Department of General Medicine, Nagasaki University Graduate School of Biomedical Sciences, Nagasaki 852-8501, Japan; 2 Department of Cardiovascular Disease Prevention, Osaka Center for Cancer and Cardiovascular Diseases Prevention, Osaka 536-0025, Japan; 3 Leading Medical Research Core Unit, Nagasaki University Graduate School of Biomedical Sciences, Nagasaki 852-8523, Japan; 4 Department of Islands and Community Medicine, Nagasaki University Graduate School of Biomedical Sciences, Nagasaki 853-0031, Japan; 5 Department of Community Medicine, Nagasaki University Graduate School of Biomedical Sciences, Nagasaki 852-8523, Japan; 6 Nagasaki University Health Center, Nagasaki 852-8521, Japan

**Keywords:** angiogenesis, HTLV-1, rs3025039, SNP, VEGF

## Abstract

Previous studies have reported a close correlation between vascular endothelial growth factor (VEGF), which plays an important role in angiogenesis, and human T-cell leukemia virus 1 (HTLV-1). However, an association between genetic characteristics related to VEGF and HTLV-1 infection has not yet been reported. Because the VEGF polymorphism rs3025039 is inversely associated with serum concentrations of VEGF, we focus on rs3025039 in the present study. To clarify the association between the VEGF polymorphism rs3025039 and HTLV-1 infection, a cross-sectional study of 1924 Japanese individuals aged 60–79 years who participated in general health check-ups was conducted. Using logistic regression, odds ratios (ORs) and 95% confidence intervals (CIs) for HTLV-1 infection in relation to rs3025039 genotype were calculated with adjustment for known confounders. Compared with rs3025039 CC-homozygotes, (T) allele carriers had a significantly lower OR for HTLV-1 infection. The adjusted OR and 95% CI for HTLV-1 infection was 0.70 (0.54–0.91) (*p* = 0.009). Genetic characteristics related to lower angiogenesis activity might be associated with a lower chance of establishing HTLV-1 infection. Although further investigation is necessary, angiogenesis might play a crucial role in the establishment of HTLV-1 infection.

## 1. Introduction

Human T-cell leukemia virus 1 (HTLV-1) spreads though parenteral, sexual, and vertical (mother-to-child) routes [1], mainly via cell-to-cell contact [2]. Therefore, the majority of HTLV-1 carriers are infected with HTLV-1 at a young age.

Most HTLV-1 carriers remain asymptomatic throughout their life [3,4,5]. However, asymptomatic HTLV-1 infection is positively associated with the progression of structural atherosclerosis among older individuals without hypertension [6]. Because the majority of older HTLV-1 carriers became infected at a young age, analysis of older individuals might enhance the understanding of the influence of asymptomatic HTLV-1 infection over the long term.

HTLV-1–infected T cells acquire the ability to secrete vascular endothelial growth factor (VEGF) [7], which is an important mediator of angiogenesis [8]. Because vasa vasorum angiogenesis is an important process that leads to structural atherosclerosis [9], individuals with asymptomatic HTLV-1 infection might have active angiogenesis.

Furthermore, angiogenesis likely contributes to the development of leukemia [10]. Increased angiogenesis and increased levels of pro-angiogenic factors have been observed in acute and chronic myeloid leukemia [11,12], myelodysplastic syndrome [12], and lymphocytic leukemia [13].

In addition, because HTLV-1 infected cells acquire the ability to secrete VEGF [7], serum concentrations of VEGF might not be influenced by genetic characteristics such as VEGF polymorphisms in HTLV-1 carriers who have a sufficient number of cells infected with HTLV-1.

Therefore, genetic characteristics that have the disadvantage of activating angiogenesis could prevent HTLV-1 activity during the early stages of HTLV-1 infection. HTLV-1 infected cells might not have increased levels of VEGF in individuals in the early stages of HTLV-1 infection. Serum VEGF concentration is reported to be inversely associated with the (T) allele of the VEGF polymorphism rs3025039 [14], which is inversely associated with structural atherosclerosis among older individuals with hypertension [15]. Because serum VEGF concentration has been reported to be positively associated with the (T) allele of the VEGF polymorphism rs3025020 [14], VEGF polymorphism rs3025020 could act as a confounder of the association between VEGF polymorphism rs3025039 and HTLV-1.

Clarifying the association between VEGF polymorphisms rs3025039 and rs3025020 and HTLV-1 infection might help to clarify the crucial role of angiogenesis during the early stage of HTLV-1 infection. We hypothesize that VEGF polymorphism rs3025039 is inversely associated with HTLV-1 infection among older individuals. To clarify the association between VEGF polymorphism rs3025039 and HTLV-1 infection, a cross-sectional study was conducted.

## 2. Materials and Methods

### 2.1. Methods

A cross-sectional study was conducted using data from the Nagasaki Island Study, which was a cohort study performed in Goto city in western Japan. The survey was conducted in different areas of Goto city over a period of three years to ensure that all areas of the city were included. Details about this survey have been provided elsewhere [16].

### 2.2. Study Population

After obtaining informed consent, 2405 Japanese individuals (922 men and 1483 women) aged 60–79 years who had undergone a comprehensive health check-up from 2016 to 2018 participated this study. Individuals without data on HTLV-1 status (n = 9), VEGF polymorphism rs3025039 genotype (n = 468), or VEGF polymorphism rs3025020 genotype (n = 4) were excluded. The remaining 1924 elderly Japanese individuals (711 men and 1213 women) were included in the analysis. Their mean (standard deviation [SD]) age was 70.3 (5.3) years.

Written consent forms were made available to ensure that the participants understood the objective of the study. The ethics committee of Nagasaki University Graduate School of Biomedical Sciences approved the study (project registration number: 14051404-13), which was conducted according to the ethical standards defined in the 1964 Declaration of Helsinki and its subsequent amendments.

### 2.3. Data Collection and Laboratory Measurements

Trained interviewers obtained medical histories from participants. Blood pressure (systolic and diastolic) was measured at rest in a seated position using a blood pressure measuring device (H-907; Omron, Kyoto, Japan). Hypertension was defined as systolic blood pressure ≥140 mmHg, diastolic blood pressure ≥90 mmHg, or antihypertensive medication use.

A chemiluminescent enzyme immunoassay kit (Fujirebio Inc., Tokyo, Japan) was used at SRL, Inc. (Tokyo, Japan) to detect HTLV-1 infection. Confirmatory tests for the detection of HTLV-1 were not performed in this study. Therefore, the presence of serum anti–HTLV-1 antibody was considered to indicate HTLV-1 infection; testing for HTLV-1 viral DNA was not performed. However, we believe that the influence of the lack of confirmatory testing for HTLV-1 infection was limited. Our previous study that involved real-time reverse transcription polymerase chain reaction with a hydrolysis probe and western blotting assays showed a low false-positive rate (1.2%) [17].

Genomic DNA was extracted from 2 mL of peripheral whole blood using a Gene Prep Star NA-480 (Kurabo Industries Ltd., Osaka, Japan). Genotyping of the single nucleotide polymorphisms (SNPs) rs3025039 and rs3025020 was conducted using TaqMan assays and a LightCycler 480 thermal cycling platform (Roche Diagnostics, Basel, Switzerland).

Carotid intima-media thickness (CIMT) was measured using B-mode ultrasonography with a LOGIQ Book XP device and a 10-MHz transducer (GE Healthcare, Milwaukee, WI, USA). Because CIMT reflects organic lesions, CIMT acts as an indicator of structural arterial stiffness. Maximum CIMT values for the left and right common carotid arteries were calculated using a semi-automated digital edge-detection software program (IntimaScope; Media Cross, Tokyo, Japan) [18].

### 2.4. Statistical Analysis

The characteristics of the study participants by rs3025039 genotype or rs2025020 genotype were expressed as means ± SD for continuous variables. The sex distribution and the prevalence of hypertension, rs3025020 or rs3025039 (CT) genotype, and rs3025020 or rs3025039 (TT) genotype were expressed as percentages. The trend test was performed in logistic regression models to evaluate *p*-values.

Logistic regression was used to calculate odds ratios (ORs) and 95% confidence intervals (CIs) to determine the association between rs3025039 genotype (three categories (CC-homozygote, CT-heterozygote, and TT-homozygote) and two categories (CC-homozygote, (T) allele carrier)) and HTLV-1 infection. In addition, logistic regression was used to evaluate the associations between HTLV-1 infection and rs3025020 genotype as a variable with three categories (CC-homozygote, CT-heterozygote, and TT-homozygote) or two categories (CC-homozygote, (T) allele carrier).

To adjust for confounding factors, two different models were conducted. Model 1 adjusted for age (years) and sex. In Model 2, we included three other potential confounding factors (hypertension (no, yes), CIMT (mm), and rs3025020 genotype or rs3025039) for the following reasons. HTLV-1 infection was revealed to be inversely associated with hypertension and positively associated with structural atherosclerosis as evaluated by CIMT [6]. Serum concentration of VEGF was reported to be inversely associated with the (T) allele of the VEGF polymorphism rs3025039 and positively associated with the (T) allele of the VEGF polymorphism rs3025020 [14]. Moreover, rs3025020 is inversely associated with hypertension [19] and rs3025039 is inversely associated with structural atherosclerosis as evaluated by CIMT among older individuals with hypertension [15].

For sensitivity analysis, we performed the main analyses for the association between rs3025039 genotype (two categories (CC-homozygote, (T) allele carrier)) and HTLV-1 infection stratified by sex.

All statistical analyses were performed using SAS for Windows, version 9.4 (SAS Inc., Cary, NC, USA). All *p*-values for statistical tests were two-tailed, and *p*-values of <0.05 were regarded as statistically significant.

## 3. Results

In this study, there were 1234 CC-homozygotes, 584 CT-heterozygotes, and 106 TT-homozygotes with the rs3025039 genotype. There were 1019 CC-homozygotes, 723 CT-heterozygotes, and 182 TT-homozygotes for the rs3025020 genotype. There were 347 HTLV-1 carriers.

### 3.1. Characteristics of the Study Population by rs3025039 Genotype

Table 1 shows the characteristics of the study participants by rs3025039 genotype (rs3025020). CT-heterozygote and TT-homozygote status for rs3025020 (rs3035039) were each significantly inversely associated with the minor allele of rs3025039 (rs3025020).

### 3.2. Association between rs3025039 Genotype (Three Categories) and HTLV-1 Infection

The association between rs3025039 genotype (three categories) and HTLV-1 infection is shown in Table 2. The minor (T) allele of rs3025039 was inversely associated with HTLV-1 infection. While the OR for TT-homozygotes was the lowest among the three categories, the difference was not statistically significant.

In the sex- and age-adjusted model (Model 1) with CC-homozygotes as the referent group, the adjusted ORs and 95% CIs for HTLV-1 infection were 0.75 (0.57, 0.97) for CT-heterozygotes and 0.59 (0.32, 1.08) for TT-homozygotes. Even after further adjustment for CIMT, hypertension, and rs3025020 genotype (Model 2), these associations remained. With CC-homozygotes as the referent group, the fully-adjusted ORs and 95% CIs for HTLV-1 infection were 0.72 (0.55, 0.95) for CT-heterozygotes and 0.57 (0.31, 1.06) for TT-homozygotes.

### 3.3. Association between rs3025039 Genotype (Two Categories) and HTLV-1 Infection

Table 3 shows the association between rs3025039 (two categories) and HTLV-1 infection. Compared with rs3025039 CC-homozygotes, (T) allele carriers had a significantly lower OR for HTLV-1 infection.

With CC-homozygotes as the referent group, the adjusted ORs and 95% CIs for HTLV-1 infection were 0.72 (0.56, 0.93) for (T) allele carriers in the sex- and age-adjusted model (Model 1) and 0.70 (0.54, 0.91) in the fully-adjusted model (Model 2).

### 3.4. Sex-Specific Analysis of rs3025039 Genotype (Two Categories) and HTLV-1 Infection

For sensitivity analysis, we performed the main analysis using sex-specific models. We found essentially the same associations. The fully adjusted ORs and 95% CIs for HTLV-1 infection among rs3025039 (T) allele carriers were 0.45 (0.27, 0.74) among men (n = 711) and 0.84 (0.61, 1.16) among women (n = 1213), respectively.

### 3.5. Association between rs3025020 Genotype (Three Categories) and HTLV-1 Infection

Table 4 shows the association between the rs3025020 genotype (three categories) and HTLV-1 infection. No significant associations between the rs3025020 genotype and HTLV-1 infection were observed.

In the sex- and age-adjusted model (Model 1) with CC-homozygotes as the referent group, the adjusted ORs and 95% CIs for HTLV-1 infection were 0.94 (0.73, 1.21) for CT-heterozygotes and 1.14 (0.76, 1.69) for TT-homozygotes. The associations were essentially the same after further adjustment for CIMT, hypertension, and rs3025039 genotype (Model 2). With CC-homozygotes as the referent group, the fully-adjusted ORs and 95%CIs for HTLV-1 infection were 0.86 (0.67, 1.12) for CT-heterozygotes and 0.96 (0.64, 1.45) for TT-homozygotes.

### 3.6. Association between rs3025020 Genotype (Two Categories) and HTLV-1 Infection

Table 5 shows the association between rs3025020 genotype (two categories) and HTLV-1 infection. No significant associations between rs3025020 genotype (two categories) and HTLV-1 infection were observed.

With CC-homozygotes as the referent group, the adjusted ORs and 95% CIs for HTLV-1 infection were 0.88 (0.69, 1.13) for (T) allele carriers in the sex and age-adjusted model (Model 1) and 0.88 (0.69, 1.13) in the fully-adjusted model (Model 2).

## 4. Discussion

The major finding of the present study involving older Japanese individuals is that VEGF polymorphism rs3025039 (T) allele carriers had significantly lower odds of HTLV-1 infection compared with CC-homozygotes. Potential mechanisms underlying the inverse association between VEGF polymorphism rs3025039 and HTLV-1 infection need to be further discussed.

Serum concentrations of VEGF are inversely associated with the (T) allele of VEGF polymorphism rs3025039 and positively associated with the (T) allele of VEGF polymorphism rs3025020 [14]. In the present study, the prevalence of the rs3025039 (T) allele carrier status was low among rs3025020 (T) allele carriers. However, the influence of the rs3025039 genotype on the association between rs3025020 allele (T) carrier status and HTLV-1 should be limited, as the associations were essentially the same in the model that did not adjust for the rs3025039 genotype (Model 1) and the model that included the rs3025039 genotype (Model 2).

Because no significant associations between the rs3025020 genotype and HTLV-1 infection were observed, the lack of angiogenesis-related hetero-cellular communication and shortage of VEGF-related tumor-induced immune suppression might play important roles in the inverse association between being an rs3025039 (T) allele carrier and HTLV-1 infection.

Angiogenesis plays a crucial role in leukemia proliferation [10]. Angiogenetic factors such as VEGF upregulate the ability of endothelial cells to make heterocellular communications with malignant hematologic cells, particularly lymphocytes infected with HTLV-1 [7]. HTLV-1 establishes cellular infection mainly via cell-to-cell contact [2]. Therefore, heterocellular communication from infected cells to non-infected cells should play a crucial role in cellular infection. Because HTLV-1 mimics VEGF to recruit heparan sulfate proteoglycans and neuropilin-1, which are involved in cellular infection, VEGF could become a selective competitor of HTLV-1 when HTLV-1 enters into non-infected cells [20]. Thus, VEGF might contribute to heterocellular communication, not direct infection, which stimulates HTLV-1 entry into non-infected cells. Further investigation is necessary in order to clarify these mechanisms.

Furthermore, VEGF contributes to tumor-induced immune suppression [21]. Therefore, individuals with genetically lower serum concentrations of VEGF might have unfavorable conditions that allow HTLV-1 to establish cellular infection. Because serum concentrations of VEGF are inversely associated with the (T) allele of the VEGF polymorphism rs3025039 [14], rs3025039 (T) allele carrier status could be inversely associated with HTLV-1 infection.

HTLV-1-infected cells secrete VEGF [7]. Therefore, proliferation of cells infected with HTLV-1 might not be associated with VEGF polymorphism rs3025039. The genetic disadvantage of elevated VEGF concentrations among (T) allele carriers could be cancelled out by HTLV-1–infected cells. However, the relationship between VEGF polymorphisms and proliferation of HTLV-1 infection among asymptomatic HTLV-1 carriers has not yet been clarified. Further investigations with larger samples and more information about VEGF and activity of HTLV-1 infected cells are warranted in order to clarify these mechanisms.

This is the first study to report that an angiogenesis-related genetic factor could influence the infectious capacity of HTLV-1. Asymptomatic HTLV-1 infection is positively associated with periodontitis [22,23]. Because disruption of the microcirculation is the main pathology in periodontitis [24], HTLV-1 infection could be associated with disruption of the microcirculation. However, the present study indicates that a genetically determined higher capacity for microcirculation maintenance might be associated with a higher risk of establishing HTLV-1 infection. Furthermore, the present findings might help to clarify the newly discovered importance of angiogenesis in establishing HTLV-1 infection and the reason that cells infected with HTLV-1 secrete VEGF [7].

Life-long follow-up studies are necessary in order to clarify the influence of symptomatic disease on the association between rs3025039 and HTLV-1 infection, as aging is a process that elevates the risk of adult T-cell leukemia-lymphoma among asymptomatic HTLV-1 carriers [25]. However, we believe that symptomatic disease has a limited influence on the present results, for of the following reasons. First, the majority of HTLV-1 carriers are infected at a young age, and establishment of HTLV-1 infection is an outcome of interest in this study. Most HTLV-1 carriers remain asymptomatic throughout their life [3,4,5]. The participants in this study were older (aged 60–79 years) and were not hospitalized.

Several limitations of the present study warrant consideration. First, there were only thirteen individuals infected with HTLV-1 who were TT-homozygotes, which might have been insufficient to detect a statistically significant association. Although an inverse association between HTLV-1 infection and the (T) allele of rs3025039 has been observed, further investigation with more individuals with HTLV-1 infection and TT-homozygote status is necessary. Second, we had no data about hematopoietic stem cells. Hematopoietic stem cells, such as CD34-positive cells, might play an important role in the present results, as CD34-positive cells contribute to the development of structural atherosclerosis [26] and progression of angiogenesis [27]. Further investigation with data on CD34-positive cells is necessary.

## 5. Conclusions

In conclusion, compared with CC-homozygotes, (T) allele carriers of the VEGF polymorphism rs3025039 have significantly lower odds of HTLV-1 infection among older Japanese individuals. Genetic factors related to a lower chance of progressive angiogenesis reduce the risk of HTLV-1 infection.

## Figures and Tables

**Table 1:** Characteristics of the study population by rs3025039 or rs3025020 genotype.

	rs3025039			*p* value
	CC	CT	TT	
No. of participants	1234	584	106	
Men, %	36.7	37.2	38.7	0.915
Age	70.4 ± 5.2	70.1 ± 5.4	69.5 ± 5.4	0.224
Hypertension, %	60.8	56.5	67.0	0.067
CIMT, mm	0.93 ± 0.2	0.93 ± 0.2	0.90 ± 0.2	0.266
rs3025020 (CC)	42.5	67.8	91.5	<0.001
rs3025020 (CT)	42.9	31.5	8.4	<0.001
rs3025020 (TT)	14.5	0.5	0.0	<0.001
	rs3025020			*p* value
	CC	CT	TT	
No. of participants	1019	723	182	
Men, %	38.3	35.1	36.8	0.408
Age	70.2 ± 5.2	70.3 ± 5.3	70.5 ± 5.2	0.693
Hypertension, %	59.9	60.7	56.0	0.516
CIMT, mm	0.93 ± 0.20	0.94 ± 0.20	0.92 ± 0.19	0.506
rs3025039 (CC)	51.5	73.3	98.4	<0.001
rs3025039 (CT)	39.0	25.4	1.6	<0.001
rs3025039 (TT)	9.5	1.2	0.0	<0.001

Values are means ± standard deviation, unless otherwise indicated. CIMT: carotid intima-media thickness.

**Table 2:** Association between rs3025039 genotype and HTLV-1.

	rs3025039				
	CC	CT	*p*	TT	*p*
Number of participants	1234	584		106	
Number of participants with HTLV-1 infection, (%)	244 (19.8)	90 (15.4)		13 (12.3)	
Model 1	Reference	0.75 (0.57–0.97)	0.031	0.59 (0.32–1.08)	0.085
Model 2	Reference	0.72 (0.55–0.95)	0.021	0.57 (0.31–1.06)	0.075

Model 1 adjusted only for sex and age. Model 2 adjusted for variables in Model 1 as well as hypertension, carotid intima-media thickness (CIMT), and rs3025020 genotype.

**Table 3:** Association between carrier of rs3025039 allele (T) and HTLV-1.

	rs3025039		
	CC	CT or TT	*p*
Number of participants	1234	690	
Number of participants with HTLV-1 infection, (%)	244 (19.8)	103 (14.9)	
Model 1	Reference	0.72 (0.56, 0.93)	0.012
Model 2	Reference	0.70 (0.54, 0.91)	0.009

Model 1: adjusted only for sex and age. Model 2 adjusted for variables in Model 1 as well as hypertension, carotid intima-media thickness (CIMT), and rs3025020 genotype.

**Table 4:** Association between rs3025020 genotype and HTLV-1.

	rs3025020				
	CC	CT	*p*	TT	*p*
Number of participants	1019	723		182	
Number of participants with HTLV-1 infection, (%)	184 (18.1)	126 (17.4)		37 (20.3)	
Model 1	Reference	0.94 (0.73, 1.21)	0.621	1.14 (0.76, 1.69)	0.526
Model 2	Reference	0.86 (0.67, 1.12)	0.265	0.96 (0.64, 1.45)	0.854

Model 1: adjusted only for sex and age. Model 2 adjusted for variables in Model 1 as well as hypertension, carotid intima-media thickness (CIMT), and rs3025039 genotype.

**Table 5:** Association between carrier of rs3025020 allele (T) and HTLV-1.

	rs3025020		
	CC	CT or TT	*p*
Number of participants	1019	905	
Number of participants with HTLV-1 infection, (%)	184 (18.1)	163 (18.0)	
Model 1	Reference	0.88 (0.69, 1.13)	0.324
Model 2	Reference	0.88 (0.69, 1.13)	0.313

Model 1: adjusted only for sex and age. Model 2 adjusted for variables in Model 1 as well as hypertension, carotid intima-media thickness (CIMT), and rs3025039 genotype.

## Data Availability

According to ethical guidelines in Japan, we cannot provide individual data due to participant privacy considerations. In addition, the informed consent obtained does not include a provision for publicly sharing data. Qualified researchers may apply to access a minimal dataset by contacting Prof. Takahiro Maeda, Principal Investigator, Department of General Medicine, Nagasaki University, Nagasaki, Japan at tamaeda@nagasaki-u.ac.jp or the Office of Data Management at ritouken@vc.fctv-net.jp. Information about data requests is also available online at: https://www.mh.nagasaki-u.ac.jp/soshin/ (accessed on7 July 2022). and http://www.med.nagasaki-u.ac.jp/cm/ (accessed on 7 July 2022).
